# Synthetic Biology-Enabled Biosensing Platforms for Point-of-Care In Vitro Diagnostics: Programmable Modules, Clinical Applications, and Translational Challenges

**DOI:** 10.3390/bios16050297

**Published:** 2026-05-20

**Authors:** Changjie Bao, Honglin Zhang, Lin Jiang, Tianhui Liu, Wei Liu, Qi Qi, Xuejiao Ren, Hongxun Fu, Meiyan Sun

**Affiliations:** 1 Academy of Laboratory, Jilin Medical University, Jilin 132013, China; 2 College of Basic Medical Sciences, Jilin Medical University, Jilin 132013, China; 3 School of Life Science, and Technology, Changchun University of Science and Technology, Changchun 130022, China; 4 College of Biomedical Engineering, Jilin Medical University, Jilin 132013, China

**Keywords:** synthetic biology, biosensing platform, point-of-care testing, CRISPR-Cas, cell-free protein synthesis, microfluidics

## Abstract

Synthetic biology is reshaping in vitro diagnostics (IVD) by enabling programmable and modular biosensing elements that can be integrated into point-of-care testing (POCT) platforms. Compared with conventional assays that depend on fixed chemistries and centralized instrumentation, synthetic biology-based systems offer adaptable molecular recognition, tunable signal processing, and flexible readout formats for decentralized diagnostics. In this review, we present synthetic biology-enabled IVD as programmable biosensing platforms organized into four functional layers: molecular recognition, signal transduction and amplification, output generation, and system integration. We discuss four major enabling modules, including cell-free protein synthesis (CFPS) systems, aptamer and riboswitch sensors, CRISPR-Cas diagnostic platforms, and microfluidic integration technologies. We summarize representative clinical applications from 2021 to 2025 in infectious disease detection, cancer biomarker analysis, and drug metabolism/toxicity screening. In addition, we examine practical considerations beyond analytical sensitivity, including matrix tolerance, workflow complexity, manufacturability, quantitative capability, and regulatory readiness. Finally, we highlight future directions for programmable diagnostics, including AI-assisted biosensor design, multimodal readouts, interoperable platform architectures, and real-world clinical validation.

## 1. Introduction

In vitro diagnosis (IVD) is a crucial cornerstone of the modern medical and healthcare system, and its core value lies in providing a vital basis for the screening, diagnosis, and treatment of diseases. Over the past half-century, IVD technology has undergone significant development, gradually progressing from relatively simple biochemical detection in the early days to a diversified analytical system encompassing high-sensitivity immunoassays and nucleic acid amplification detection. Classic methods such as enzyme-linked immunosorbent assay (ELISA) and polymerase chain reaction (PCR), with their precise quantitative capabilities for proteins and nucleic acids, have become the “gold standards” in many clinical testing scenarios [1,2,3]. However, these classic methods often rely on traditional laboratories, expensive instruments, and operation by professional technicians. This dependence not only drives up the cost of a single test but also restricts the expansion of such testing services in resource-poor areas. Therefore, the current IVD infrastructure, due to its complex system, is difficult to meet the current demands for rapid, decentralized, and patient-centered diagnosis. This contradiction is particularly prominent in scenarios such as point-of-care testing (POCT), community screening, and home health monitoring.

At the same time, synthetic biology stood out as a rapidly developing interdisciplinary field, integrating molecular biology, systems biology, bioinformatics, and engineering design. Its core goal is to transform life systems into programmable substances by defining standardized biological components, assembling them into energy modules, and arranging these modules into advanced systems with predictable and controllable behavior [4,5,6]. Milestone advances such as minimal genome design, synthetic cells, modular gene circuits, and automated biomanufacturing workflows have reshaped the fields of metabolic engineering, biomanufacturing, and environmental restoration [7,8,9,10,11,12]. Synthetic biology has the characteristics of programmability, modularity, and cell-free compatibility. These key features directly address the primary challenges of IVD, including the need for customizable sensing, robust signal processing, low-cost production, and applications beyond the central laboratory.

In recent years, these two development paths have begun to meet more clearly. A series of synthetic biology modules, including RNA-based toehold switches, aptamer and riboswitch sensors, CRISPR-Cas nucleic acid testing systems, and cell-free protein synthesis (CFPS) platforms, have been integrated into paper-based devices, lateral flow detection, microfluidic control chips, and emerging wearable systems [13,14,15,16,17]. In these devices, synthetic components are directly embedded in the diagnostic workflow as programmable identification and calculation units. This makes it possible to visualize readings, instrument lighting operations, and logical-gate control decision-making. For example, in the field of infectious disease diagnosis, synthetic biology enables rapid nucleic acid testing under resource-scarce conditions, with a turnaround time of less than 30 min and minimal equipment requirements [18,19,20]. In oncology, synthetic circuits have been used specifically to detect multiple tumor-related biomarkers. In the field of drug development, synthetic biosensors and CFPS-based platforms have significantly accelerated the development of new drugs and toxicity screening [19,21,22].

This review is organized around four principal themes. First, we summarize the fundamental principles of synthetic biology and review the evolution of IVD technology, elucidating their inherent convergence. Second, we dissect synthetic biology-enabled IVD into four core modules: CFPS systems, nucleic acid sensors, CRISPR-Cas platforms, and microfluidic integration. And we systematically examine their respective roles across the diagnostic layers of molecular recognition, signal transduction, output generation, and system integration. Third, we showcase representative applications in infectious disease detection, cancer biomarker analysis, and drug metabolism/toxicity screening. Finally, we critically evaluate key translational challenges, including standardization, manufacturability, ethical considerations, and regulatory pathways, while delineating future directions. Our objective is to provide a comprehensive framework to facilitate the practical deployment of synthetic biology-based diagnostics.

## 2. Basic Concepts and Development of Synthetic Biology

### 2.1. Definition and Core Concepts

The definition of synthetic biology typically focuses on “the rational reconstruction and functional customization of biological systems.” This goal is achieved through the design of standardized components, modular assembly, and engineering control. The ultimate pursuit in this field is to make biological processes predictable, controllable, and even designable at the theoretical level [23]. The field of synthetic biology has drawn on the concept of hierarchical abstraction in electronics to construct a system architecture comprising three core levels: biological components, functional modules, and integrated systems.

Biological components are fundamental units that encompass DNA regulatory elements, coding sequences, and protein domains, among other components. All these components are designed to be reusable. Functional modules are “functional units” formed by assembling the above components and can independently perform perception, processing, and signal driving, etc. An integrated system is an extensive network composed of multiple functional modules that supports complex biological behaviors, such as decision-making and memory formation [7,8,9].

In the practical application of synthetic biology, three design principles are particularly crucial. The first is modularization, which breaks down complex functions into relatively independent units. This not only simplifies the design process but also enables these units to be easily reused in new application scenarios. The second is distributed regulation, which introduces feedback loops and interaction networks to eliminate the need for continuous external intervention. This greatly enhances the robustness and environmental adaptability of the system. The third principle is the self-organization. By leveraging the inherent interactions between molecules, it can function independently without requiring precise regulation of microscopic processes. These design principles provide crucial support for integrating synthetic biology and IVD technology.

These principles are highly consistent with the needs of IVD. Modularization and abstraction facilitate the construction of a diagnostic loop, enabling quick retargeting to new biomarkers by simply replacing the sensing elements. Distributed regulation enables logic and threshold functions to be directly embedded in the diagnostic module. On the other hand, self-organization enables complex signal-processing behaviors to emerge from relatively simple molecular building blocks. In summary, these features provide a conceptual basis for the transformation from a fixed-function diagnostic kit to a truly programmable diagnostic system.

### 2.2. Top-Down and Bottom-Up Technology Pathways and Relevance to IVD

At present, the development of synthetic biology primarily follows two complementary technical paths, often referred to as top-down and bottom-up methods.

The top-down method takes natural cells as the chassis, and then transforms them through genome minimization or targeted engineering. A well-known example is the smallest bacterial genome reported by Hutchison and his colleagues, which contains 473 genes but can still support controlled growth and division [10]. By removing redundant or under-researched genes, this minimalist cell can provide a relatively pure background, reducing endogenous noise and thereby improving the predictability of the synthetic gene loop. Theoretically, these streamlined cell models can support the diagnostic application of living cells. For example, engineered intestinal bacteria are utilized to detect disease-related metabolites, while probiotic biosensors are employed to report inflammation [21,24]. Their reduced complexity and improved genetic stability may help to increase the robustness of cell-based diagnostics in fluctuating physiological and environmental conditions.

The bottom-up synthesis pathway starts with non-biomolecular building blocks, such as nucleic acids, proteins, and lipids. It uses these building units to construct synthetic cells or primary cells that show the characteristics of specific types of life. Minagawa et al. developed self-growing protocell models in an aqueous two-phase poly(ethylene glycol) (PEG) and dextran (DEX) system, in which transcription-translation (TXTL) reactions and DNA replication sustained continuous gene expression and compartmental expansion, thereby providing a model of early life-like behavior [11]. Hartmann et al. created a programmable platform for in vitro biocomputing. They achieved this by designing orthogonal light-activated DNA modules and encapsulating them in synthetic cells. This design enables precise spatiotemporal control over the expression of RNA and protein [12].

For IVD, the bottom-up research and development path is particularly important because it supports the development of cell-free systems and synthetic compartments, which can carry diagnostic circuits without being limited by living cells. After CFPS is freeze-dried on paper or in micro-flow control chips, it is essentially a miniature biochemical factory that can convert molecular input into protein output as needed. Similarly, liposomes, hydrogels, and related synthetic compartments provide controlled microenvironments for modular diagnostic reactions. Although top-down approaches point toward future live-cell diagnostic tools, most current synthetic biology-enabled IVD platforms rely on bottom-up modules, including CFPS, synthetic nucleic acid sensors, and CRISPR-based systems.

In general, the interaction between top-down and bottom-up strategies has deepened our understanding of the principles of biological design and accelerated the transformation of synthetic systems into practical applications, including diagnosis.

## 3. Brief History of In Vitro Diagnostic Technology and Convergence with Synthetic Biology

**Figure 1:**
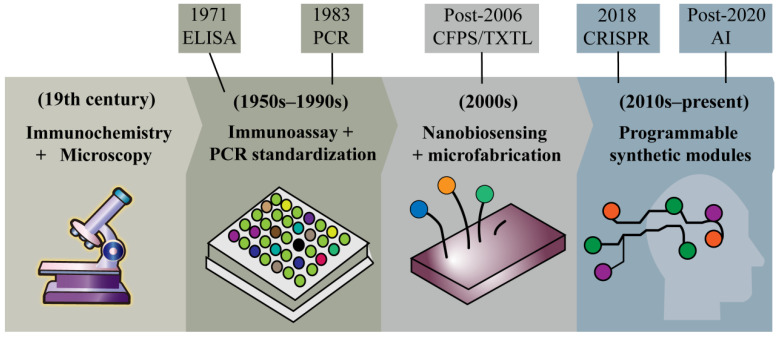
The development of IVD.

IVD technology can be roughly divided into four stages of development. Each stage introduced important functions, but also exposed bottlenecks, which eventually prompted the introduction of synthetic biology (Figure 1).

(i) Immunochemistry and microscopy milestone (19th century). Early IVD mainly depends on the qualitative analysis of body fluids. Bright’s description of kidney disease through proteinuria in 1827 and Snow’s roadmap for cholera transmission through fecal analysis in 1854 are often regarded as typical cases. At that time, the diagnosis was mainly based on visual measurement and simple chemical reactions. As expected, the sensitivity and specificity of these methods are very low and highly dependent on operator skill, underscoring the need for standardized biochemical detection methods.

(ii) Immunoassay standardization and nucleic-acid amplification milestone (mid-20th century to 1990s). Advances in immunology and molecular biology have ushered in an era of greater standardization in IVD. The development of ELISA has made it possible to quantitatively detect protein biomarkers, such as hormones and antibodies, while PCR has completely changed the way nucleic acid testing is conducted by significantly improving sensitivity [25]. Together, these technologies form the cornerstone of modern centralized diagnosis. At the same time, they have introduced new bottlenecks, including dependence on cold-chain stabilization reagents, precision instruments, and well-trained personnel, thereby increasing costs and limiting service availability.

(iii) Microfabrication and advanced biosensing milestone (2000s). Nanotechnology, photonics, and advanced biosensing techniques extended IVD performance beyond what traditional immunoassays and PCR could provide. Quantum dot labeling enabled detection limits in the femtomolar range, and surface plasmon resonance (SPR) enabled label-free, real-time measurement of binding interactions [26,27,28]. These innovations substantially improved sensitivity and throughput. However, they often increased system complexity and cost and did not, by themselves, resolve challenges related to portability or programmability.

(iv) Programmable synthetic modules milestone (2010s-present). The integration of synthetic biology modules, wearable electronics, and artificial intelligence is now steering IVD toward decentralized, intelligent, and user-centric systems [24]. Synthetic biology effectively reduces the dependence on natural enzymes and antibodies by providing programmable nucleic acids and protein components. These components have modifiable flexibility. Even for new detection targets, they can be quickly adapted with only a small optimization. At the same time, microfluidic technology enables the integration of fluid-processing automation and detection, and an artificial intelligence algorithm further enhances the efficiency and accuracy of signal interpretation. The three work together to make IVD technology more efficient and flexible [29]. In the following chapters, we will formalize the integration of synthetic biology and IVD by introducing a theoretical framework, and then dissect the core modules that drive contemporary IVD innovation.

## 4. Theoretical Basis for Combining Synthetic Biology with IVD

**Figure 2:**
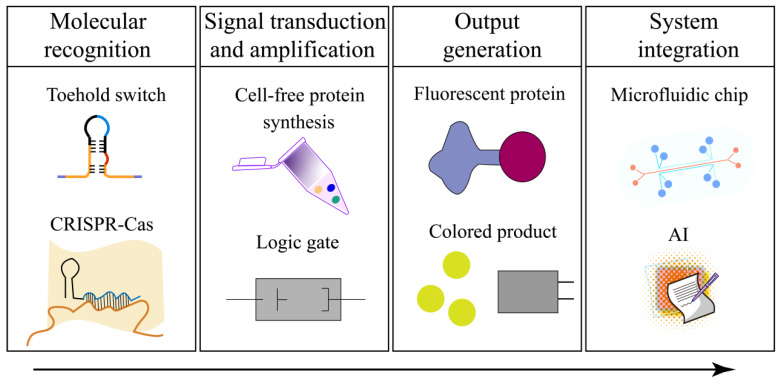
Theory for integrating synthetic biology with IVD.

The diagnostic workflow can be abstracted as a programmable signal-processing chain consisting of four functional layers: (1) molecular recognition, (2) signal transduction/amplification, (3) output generation, and (4) system integration and decision making (Figure 2). Synthetic biology provides standardized, reconfigurable building blocks for each layer.

At the recognition layer, programmable nucleic-acid interactions and guide-directed enzymes provide target specificity; at the transduction/amplification layer, biochemical reactions convert recognition into amplified measurable outputs; at the output layer, diverse reporters enable context-appropriate readouts (e.g., fluorescence, colorimetric, electrochemical); and at the integration layer, microfluidic or paper-based formats support automation, multiplexing, and closed-workflow operation.

This abstraction is useful because it separates what a diagnostic system must accomplish from how each function is implemented, enabling systematic comparison of modules, explicit discussion of trade-offs, and rational design of integrated sample-to-answer platforms.

## 5. Core Technologies Enabling Synthetic Biology-Based IVD

After establishing the conceptual framework, we now turn to four core technological modules: CFPS systems, nucleic acid sensors and switching elements, CRISPR-Cas systems, and microfluidic integration. Building upon the signal-processing framework in Section 4, we next examine each enabling module in depth, focusing on operating principles, performance trade-offs, and integration constraints relevant to real-world IVD deployment.

### 5.1. CFPS Systems

**Figure 3:**
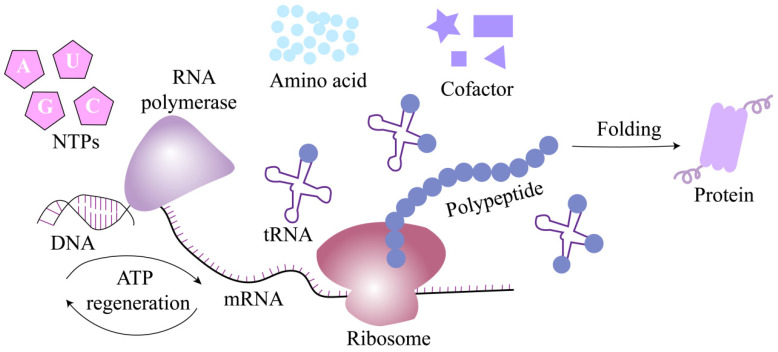
Schematic diagram of the CFPS working principle. Note: DNA serves as the template for mRNA synthesis, a process catalyzed by RNA polymerase, which utilizes NTPs as substrates. Subsequently, mRNA is translated into a polypeptide chain by ribosomes, with the involvement of tRNA, amino acids, cofactors, and ATP regeneration systems. The newly synthesized polypeptide then undergoes folding to assume its functional protein conformation.

CFPS is usually based on crude extracts or purified components from prokaryotic or eukaryotic cells (such as *Escherichia coli*, Chinese hamster ovary, wheat germ, etc.) to drive transcription and translation in vitro [30]. By decoupling protein synthesis and cell growth, the system offers several advantages over traditional cell expression methods, many of which are highly consistent with the needs of IVD. With the help of the CFPS system, protein preparation can be completed in a few hours, effectively eliminating the time-consuming steps of cloning, transformation, and cell culture in traditional methods, and significantly improving the efficiency. The reaction composition (including DNA templates, salts, cofactors, and energy) is fully adjustable, allowing fine optimization (Figure 3). Since there are no living cells, biosafety considerations and downstream integration have been simplified. Additionally, CFPS reagents can be freeze-dried onto paper or chips and then rehydrated with water or a small amount of treated sample [31,32].

In the IVD empowered by synthetic biology, CFPS plays two significant roles. Firstly, it facilitates the rapid production of diagnostic reagents. Gupta et al. successfully produced the SARS-CoV-2 receptor-binding domain (RBD) using the ALiCE eukaryotic CFPS system derived from tobacco cells and achieved correct glycosylation and disulfurization. This kind of work demonstrates the feasibility of rapidly generating structurally complex antigens for vaccines and serological detection [33]. Ramm et al. used mammalian lysate CFPS to express cholera toxin and a thermostable enterotoxin, confirming correct polymerization and cytotoxic activity, thereby providing a safe platform for the production of toxin antigens and control products [34]. Taguchi and Niwa reviewed recombinant CFPS as a tool for studying protein folding and aggregation, and proposed insights to guide the design of robust diagnostic enzymes and reporter proteins [35]. Compared with traditional cell culture, CFPS shortens the research and development cycle, facilitates the expression of toxic or unstable proteins, and allows rapid iterative antigen design. However, the cost of the reagent for each reaction may be higher.

Secondly, CFPS can serve as a signal transduction and amplification module within the diagnostic loop. Kocalar et al. have verified the CFPS on the International Space Station, demonstrating that the BioBits reaction can synthesize RNA adapters and fluorescent proteins, and construct biosensors sensitive to small molecules and RNA sequences under space conditions [31]. Soltani and Bundy designed CFPS biosensors that can operate in human saliva, serum, and urine by co-expressing RNase inhibitors during extract preparation, thereby significantly reducing detection costs while maintaining performance [36]. Park et al. developed a CRIVER system that couples CRISPR-Cas collateral cleavage with CFPS, enabling programmable release of DNA triggers to activate reporter protein production for sensitive and multiplexed detection of both RNA and DNA targets [37]. These studies emphasize that CFPS is a multifunctional biochemical engine that converts the input nucleic acid detected by the toehold switch or aptamer into an amplified protein output signal.

Compared with the enzyme-based detection method, which relies solely on purified proteins and fixed reaction schemes, the CFPS-based platform is reconfigurable, as the new detection module can be reconstructed by replacing the DNA template. They also integrate naturally with synthetic circuits, enabling on-demand production of reagents. At the same time, the CFPS reaction is more sensitive to temperature, ion strength, and batch-to-batch differences in extract preparation [38,39]. From an engineering perspective, CFPS performance is governed by coupled kinetic and physicochemical parameters rather than by module identity alone. Key determinants include transcription and translation initiation rates, Mg^2+^ and K^+^ concentrations, energy regeneration efficiency, viscosity, macromolecular crowding, and the abundance of inhibitory species introduced by the sample matrix. Unlike living cells, CFPS lacks homeostatic buffering; therefore, even modest shifts in ionic strength, pH, or nuclease burden can disproportionately alter reaction trajectories, reporter accumulation, and background noise. Batch-to-batch variability further changes extract composition, ribosome activity, and enzyme balance, thereby reducing reproducibility across laboratories. For this reason, robust CFPS-based diagnostics should be designed not only by selecting sensing elements and reporters, but also by quantitatively controlling reaction-state variables through extract standardization, formulation engineering, the use of stabilizing additives, and design-of-experiments optimization. The current improvement methods for CFPS, including experimental design optimization, stabilizing additives, and optimized freeze-drying strategies, aim to enhance the robustness and shelf life of the products [32,40].

### 5.2. Nucleic Acid Sensors and Switching Elements

Nucleic acid sensors, such as Toehold switches, aptamers, and riboswitches, serve as programmable recognition and regulation elements, converting molecular binding events into changes in gene expression or reporter activity. Here we will discuss this in two parts.

#### 5.2.1. Toehold Switches for RNA, miRNA, and Pathogen Detection

**Figure 4:**
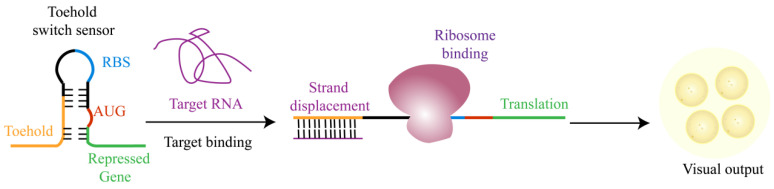
Working principle of the toehold switch sensor. Note: The target RNA specifically binds to the hold domain, initiating strand displacement and consequently exposing the ribosome binding site (RBS) along with the translation initiation codon AUG. This process activates translation of repressed genes, ultimately generating visual output signals.

The toehold switch is the synthetic RNA structure that controls translation initiation. A typical design includes a 5′ toehold domain, a stem-ring structure that separates the ribosome binding site (RBS) and the start codon, and a downstream reporter gene [41]. In the absence of the target RNA, the stem loop blocks translation. When the target RNA recognizes the toehold domain and undergoes base complementarity, the stem loop unfolds, exposing the RBS and start codon, thereby initiating the translation of the reporter gene. In this way, when the target RNA is present, a recognizable signal will be expressed (Figure 4).

Recent related research has expanded the target range and improved the performance of the toehold switch in IV. Morey et al. combined a toehold switch targeting the SARS-CoV-2 nsp2 region with an amplification module based on tobacco etch virus (TEV) protease to achieve fmol-level detection without prior nucleic acid amplification, and demonstrated rapid detection [42]. Ekdahl et al. engineered toehold-mediated switches for the in vivo detection of native bacterial RNAs, indicating their potential for live-cell sensing [41]. Copeland and Kwon assessed toehold switches coupled to the exponential amplification reaction (EXPAR) for microRNA detection in CFPS. They concluded that the combined system can yield high sensitivity and specificity suitable for miRNA biomarker diagnostics [43]. Kang et al. developed a LuxSit-i-enhanced toehold switch assay for *Vibrio parahaemolyticus* that uses recombinase polymerase amplification (RPA) or nucleic acid sequence-based amplification (NASBA) to distinguish viable from non-viable bacteria, a property that is particularly attractive for POCT in food safety [44].

The performance of toehold switches is similarly dictated by biophysical parameters that determine the balance between repression and activation. Leakiness arises when the OFF-state hairpin is insufficiently stable and transiently exposes the ribosome-binding site or start codon, whereas poor sensitivity may result when the trigger RNA is buried within stable secondary structures or competes with alternative intramolecular folds. In practical terms, switching behavior depends on the free-energy difference between OFF and ON conformations, the on/off rates of hybridization, trigger accessibility, and operating temperature. These considerations explain why a design that performs well in a buffer may lose discrimination in complex matrices or under field conditions. Accordingly, future optimization should combine sequence-level design, thermodynamic screening, and machine-learning-assisted prediction with experimental characterization of leakage, dynamic range, response time, and mismatch tolerance, thereby moving toehold switches closer to standardized and predictive engineering [45,46].

#### 5.2.2. Aptamers and Riboswitches for Protein and Small-Molecule Biomarkers

An aptamer is a single-stranded DNA or RNA molecule selected to bind specifically to targets, including proteins, peptides, and small molecules, and to exhibit high affinity and specificity. When integrated into specific nucleic acid structures, aptamer binding can trigger conformational changes, thereby regulating the reporter activity and forming the basic components of biosensors.

Tripathi et al. isolated a novel ssDNA aptamer (Kd = 166 nM) of the anti-ovarian cancer biomarker CA125. They applied it to a laterally flowing band, demonstrating its concentration-dependent detection and potential for early cancer screening [47]. Muhammad et al. designed an RNA aptamer targeting the tyrosine kinase domain of the NT-3 growth factor receptor using computational sequence modeling and molecular docking, obtaining a candidate with a favorable binding energy (MFE = −32.2 kcal/mol) for cancer diagnosis and treatment [48].

Riboswitch-like constructs can be created by coupling protein-binding aptamers to translation-regulatory elements. Vezeau et al. [49] developed an automated pipeline that converts protein-binding RNA aptamers into riboswitches that control translation in CFPS. The resulting sensors responded to human biomarkers such as C-reactive protein (CRP) and interleukin-32γ (IL-32γ), with dynamic ranges up to 16-fold over clinically relevant concentration ranges. Beyond conventional aptamer switches, photoactivatable aptamers have recently been engineered to achieve precise spatiotemporal control via light-responsive chemical groups or photosensitive nanomaterials, thereby significantly improving the selectivity and anti-interference capability of point-of-care testing platforms [50].

Aptamer and ribose-switch sensors complement toehold switches by enabling the detection of non-nucleic acid substances. However, at different salt concentrations and temperatures, their performance is affected by matrix composition, off-target effects, and structural instability. Therefore, it is urgently necessary to establish standardized characterization methods and design rules.

### 5.3. CRISPR–Cas Systems Integrated with In Vitro Detection

**Figure 5:**
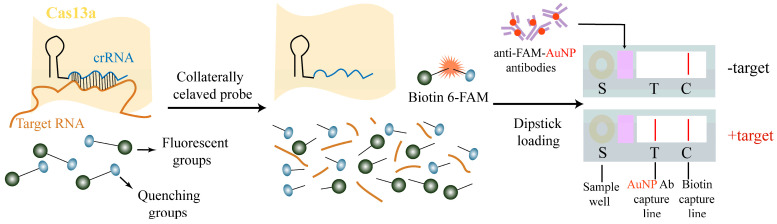
Schematic diagram of the Cas working principle. Note: The Cas13a/crRNA complex binds to the target RNA, thereby activating the collateral cleavage activity of Cas13a, which cleaves the reporter probe containing 6-FAM and biotin, and subsequently displays the results on the lateral flow test strip.

The CRISPR-Cas system was initially regarded as an adaptive immune mechanism of bacteria and archaea. Due to their high efficiency in gene-editing technology, they have been repositioned as a highly sensitive and specific nucleic acid detection platform [51]. Effector proteins such as Cas12a and Cas13a exhibit “collateral cleavage”. Once activated by the binding of guide RNA (crRNA) to the target sequence, they will activate their potent nuclease activity, cutting the surrounding single-stranded DNA or RNA, thereby separating the fluorescent group from the quencher group and exposing a recognizable detection signal (Figure 5).

Li et al. [52] developed a Cas13a-based SHERLOCK (specific high-sensitivity enzymatic reporter unlocking) assay for Crimean–Congo hemorrhagic fever virus (CCHFV), using degenerate crRNA sets to accommodate viral sequence variability. Their platform achieved a detection limit of one copy per microliter. It discriminated among viral lineages without cross-reactivity, demonstrating how CRISPR diagnostics can be designed to accommodate genetic diversity in emerging pathogens. Chen et al. [53] introduced a split-crRNA Cas12a system that enables the amplification-free detection of miRNAs, such as miR-21, and DNA targets. They demonstrated the multiplexed detection of HPV subtypes in plasma from cervical cancer patients. Yoshimi et al. [54] proposed a CRISPR-Cas3-based CONAN (Cas3-operated nucleic acid detection) platform for detecting SARS-CoV-2 and influenza viruses, exploiting Cas3’s processive DNA degradation for signal amplification and enabling discrimination of single-nucleotide variants. Overall, CRISPR-based biosensors have revolutionized analytical chemistry by enabling specific detection of nucleic acid and even non-nucleic acid targets, and their integration with isothermal amplification and microfluidic/portable devices has successfully translated them into practical POC solutions [55].

CRISPR-based diagnostics can be divided into two categories: amplification-dependent systems and non-amplification-dependent systems. Amplication-dependent systems, for instance, those that combine RPA or LAMP (loop-mediated isothermal amplification) with CRISPR, offer very low detection limits but increase complexity and risk of contamination. The system without amplification simplifies the workflow, but it typically exhibits a higher detection limit and lower accuracy than the amplification-dependent system. Currently, most CRISPR diagnostics can only yield semi-quantitative or qualitative results, such as the appearance of lines on lateral flow strips. Therefore, quantitative electrochemistry and digital CRISPR readings are future directions for development [56].

Many CRISPR diagnostic platforms have reached or approached the standards set by the World Health Organization (WHO), which emphasize rapidity, specificity, and stability. Despite this, several significant challenges remain, including contamination control in amplification systems, batch-to-batch variability in enzyme preparations, and the difficulty in achieving precise quantification. The regulatory pathways for CRISPR diagnosis are still evolving [57].

### 5.4. Microfluidic Integration with Synthetic Modules

Due to the laminar flow of fluids in microscale channels, microfluidic chips can precisely control reaction time, mixing concentration, and reaction temperature using different components, while reducing reagent consumption. In synthetic biology, microfluidic platforms offer significant advantages for building compact, automated diagnostic devices.

Tonooka reported the first freeze-dried CFPS system in polydimethylsiloxane (PDMS)-glass microchambers, demonstrating that lyophilized reactions could be stored and subsequently rehydrated to produce proteins on-chip [58]. Compared with paper-based CFPS, freeze-drying using microchambers significantly enhances its uniformity and stability, and the system demonstrates plug-and-play diagnostic capabilities for detecting N-acyl-hyperserine lactone (AHL). Zhang et al. described an integrated device that combines aerosol sampling with a microfluidic LAMP–CRISPR chip for real-time monitoring of airborne viruses, including SARS-CoV-2, thereby demonstrating the feasibility of environmental surveillance [59].

Microfluidics can also integrate workflows by combining sample lysis, nucleic acid amplification, CRISPR detection, and reading in a single device. It enhances the reliability of the test results by standardizing the control of fluid volume, reaction time, and temperature curves. Meanwhile, it is also easy to operate, allowing users to add samples, start automatic sequences, and obtain readings through the built-in optical module. Common examples, such as blood glucose meters and nucleic acid testing devices for SARS-CoV-2, are typical combinations of microfluidics and IVD.

To systematically compare the performance differences among the above-mentioned core synthetic biology-enabled in vitro diagnostic technologies and clarify the application advantages and applicable scenarios of each technology, the key performance indicators for each technology are summarized below (Table 1). Importantly, no single synthetic biology-enabled module is uniformly superior across all diagnostic scenarios. Analytical sensitivity is only one dimension of performance; cost per test, workflow complexity, tolerance to crude samples, ease of multiplexing, quantitative accuracy, manufacturability, and regulatory maturity are equally important for real-world deployment. Thus, strategic technology selection should be based on clinical context rather than the absolute limit of detection alone. In some use cases, a moderately sensitive but highly portable and low-cost platform may be preferable to an ultrasensitive assay that requires complex upstream processing or specialist operation.

## 6. Typical Application Cases

Synthetic biology-based IVD platforms have been applied in a wide range of scenarios. Here, we group representative examples into three categories that correspond to primary clinical needs: rapid infectious disease detection, cancer-related biomarker testing, and drug metabolism and toxicity screening. For each category, we highlight how synthetic modules overcome specific limitations of conventional diagnostics.

### 6.1. Rapid Detection of Infectious Diseases

Rapid detection of infectious diseases is a priority area in which high sensitivity, short turnaround times, and minimal infrastructure are all crucial. Synthetic biology contributes by embedding programmable recognition and amplification modules into portable formats that often align well with WHO ASSURED criteria.

In nucleic acid-based diagnosis, Kang et al. developed a cell-free toehold switch assay for hepatitis A virus type I (HAV I), which uses the conserved VP1 gene as the target and achieves visible green fluorescence detection with a total sample-to-result time of approximately 3 h (including 2 h of NASBA amplification and 1 h of cell-free expression), or approximately 45 min for the detection step alone following NASBA, using heat-treated viral entities or in vitro-transcribed RNA as inputs, though it may be susceptible to nuclease interference when applied directly to complex matrices such as untreated fecal or water samples without prior nucleic acid purification [60]. Chen et al. developed a magnetic separation-enhanced colorimetric biosensor (termed C-TEC) for *Mycobacterium tuberculosis* (MTB) detection, which uses the 16S rDNA as the target and achieves visual detection by the naked eye down to 50 fM or UV–vis detection at 520 nm down to 20.71 fM within 90 min, through a toehold-containing three-way junction (TWJ)-initiated multiple EXPAR coupled with CRISPR/Cas14a-assisted trans-cleavage and gold nanoparticle (AuNP)-based colorimetric signal readout, though the system may require careful optimization of primer and template concentrations to minimize false-positive signals arising from nonspecific amplification [61].

CRISPR-based diagnosis further expands the opportunities for detecting infectious diseases. Zhu et al. developed a CRISPR-Cas13a-based assay coupled with RPA for the rapid detection of the *mexX* efflux pump gene in *Pseudomonas aeruginosa*, which uses the *mexX* gene as the target and achieves fluorescence-based detection with limit of detection (LOD) as low as 1 aM for the two-step format and 10 aM for the one-tube format, with total sample-to-result times of 40 min (two-step) and 5 min (one-tube), respectively, and also enables lateral flow dipstick-based visual detection with an LOD of 10 fM, though the one-tube format exhibits slightly lower sensitivity compared to the two-step format due to potential incompatibility between RPA and CRISPR reagents in a single reaction [62]. You et al. developed a portable Cas12a-based nucleic acid detection platform combined with up-converting phosphor technology lateral flow assay (Cas12a-UPTLFA) for the rapid detection of *Yersinia pestis*, which uses the plasmid-borne pla gene as the primary target and achieves a limit of detection as low as 3 aM for purified genomic DNA and 10^2^ CFU per 100 μL of spiked mouse blood within approximately 1.3 h (including 18 min RPA, 45 min Cas12a reaction, and 15 min UPT-LFA readout) with a portable biosensor, though the assay showed relatively higher interference in lung tissue samples (detection limit of 10^5^ CFU per 20 mg) compared to blood or soil matrices, and its sensitivity (93.75–95.83%) and specificity (90.63–96.88%) in spiked blood samples were slightly lower than those of the fluorescence-based RPA-Cas12a readout and qPCR [63].

Synthetic protein sensors offer a complementary approach. Devoy et al. developed a modular protein protease sensor platform comprising a GFP backbone with N-terminal Flag and C-terminal His tags separated by a cleavable linker, which uses a protease-specific cleavage sequence as the recognition element and enables quantitative detection via sandwich ELISA (LOD ~10 ng) or fluorescence readout (up to 5.5-fold after 1.5 h) following tag uncoupling, validated for TEV, SARS-CoV-2 Mpro, and cancer-associated MMP9; however, the fluorescence approach showed a ~45 min maturation delay and lower efficiency than ELISA, and the MMP9 sensor yielded a non-significant reduction (14%, *p* = 0.2719), indicating a need for optimizing cleavage accessibility and tag selection for certain proteases [64].

Taken together, the principal advantage of synthetic biology-enabled infectious disease diagnostics is not simply that some systems reach attomolar or femtomolar sensitivity, but that they combine programmability with deployability. Compared with RT-qPCR, these platforms can reduce instrument dependence, shorten hands-on workflow, and facilitate rapid retargeting to emerging pathogens or resistance markers. Compared with antigen tests, they generally offer higher molecular specificity and more flexible signal amplification architectures. However, these gains are accompanied by trade-offs: amplification-assisted CRISPR or toehold-based assays may introduce additional workflow steps and a higher risk of contamination; many platforms remain semi-quantitative rather than fully quantitative; and regulatory maturity still lags behind established PCR-based assays. Therefore, their most compelling role at present may be in decentralized, rapid-response settings where adaptability and low infrastructure requirements outweigh the need for absolute quantification.

### 6.2. Cancer-Related Biomarker Testing

Cancer diagnostics pose particular challenges because they require detecting low-abundance biomarkers, such as microRNAs and circulating tumor DNA, and interpreting complex patterns across multiple markers and spatial contexts. Synthetic biology platforms contribute both conventional IVD-type assays and cell-based reporter systems that complement imaging and liquid biopsies.

In cell-free and nanodevice-based diagnostics, Li et al. developed DNA tetrahedron-based logic nanodevices (TDLNs) with localized catalytic hairpin assembly for the sequential imaging of membrane MUC1 and cytoplasmic miR-21, achieving detection limits of 201 pM for MUC1 and 28.3 pM for miR-21 within approximately 1 h for AND-gate identification of cancer cells, though the requirement for dual-marker co-expression may limit its application in cells with low or heterogeneous biomarker levels [65]. By encoding logical relationships between markers, Tripathi et al. improve specificity and support more nuanced stratification of cancer cell populations. Aptamer-based lateral-flow and electrochemical sensors targeting cancer biomarkers such as CA125, HER2, and PD-L1 provide additional pathways for low-cost screening [47].

In cell-based diagnostic systems, Przybyszewska-Podstawka et al. developed split-Cas9-based AND logic gates with intein-mediated reconstitution for detecting cellular events, using conditional promoters (e.g., hCEA, TWIST1-BD) or cell–cell fusion as inputs to activate an EGxxFP reporter, achieving specific readouts in epithelial cancer cells, undergoing EMT, or syncytia formation within 1–3 days, though reliance on promoter activity and fusion efficiency may limit sensitivity in heterogeneous or low-expression contexts [66]. Shin et al. developed an antigen-inducible SNIPR-based T cell reporter system for PET imaging of breast cancer and glioblastoma, which uses HER2 or EGFRvIII as the target and achieves >10-fold higher [^18^F]FHBG accumulation in antigen-positive tumors versus controls in dual-xenograft models within 8–10 days post T cell injection, though the signal decreases after day 10 due to limited target killing by CD4^+^ T cells [67]. Although SNIPR is not a conventional in vitro test, it demonstrates how synthetic circuits can transform immune cells into living diagnostic reagents, enabling the integration of diagnosis and treatment.

In oncology, the distinctive contribution of synthetic biology is the ability to encode molecular logic, integrate multiple weak signals, and detect dynamic cellular states that are difficult to capture with conventional single-analyte assays. Relative to standard immunoassays, these systems can improve specificity by requiring combinatorial biomarker inputs rather than relying on a single elevated marker. Relative to imaging alone, they offer the possibility of molecularly resolved readouts and earlier detection of biochemical changes. Nonetheless, these advantages must be weighed against current limitations in assay standardization, throughput, quantitative reproducibility, and clinical validation across heterogeneous patient cohorts. Accordingly, the near-term translational opportunity lies less in replacing established pathology or imaging workflows outright than in complementing them with multiplexed liquid-biopsy-compatible tools that improve risk stratification, monitoring, and decision support.

### 6.3. Drug Metabolism and Toxicity Screening

Synthetic biology also contributes to preclinical drug metabolism and toxicity screening, which can be considered a broader class of functional diagnostics that inform therapeutic choices and safety assessments.

At the level of target identification and validation, Zhang et al. developed a yeast two-hybrid-based screening system to identify inhibitors of the FtsZ/SepF interaction in *Mycobacterium tuberculosis*, leading to compound T0349 that binds specifically to SepF (KD ≈ 47.7 μM) and blocks FtsZ/SepF interaction, achieving anti-Mtb MICs of 1–4 μg/mL against drug-sensitive and multidrug-resistant strains with low toxicity to other bacteria and mice (LD50 > 500 mg/kg), though the compound shows suboptimal activity compared to first-line drugs and requires structural optimization [68]. Such synthetic biology-enabled workflows accelerate the discovery of both diagnostic and therapeutic targets. At the resistance-profiling level, the CRISPR-Cas13a/RPA assay for *mexX*, as discussed above, provides a rapid functional diagnostic that can guide rational antibiotic use [62].

At the toxicity screening level, Park et al. [69] evaluated five traditional Oriental medicines (TOMs) for tau pathology, using okadaic acid-induced hyperphosphorylation, tau K18 aggregation/dissociation (ThT assay), and tau-induced neurotoxicity/inflammation in HT22 and BV2 cells, finding that Hwanglyeonhaedoktang (HLT) most effectively inhibited tau hyperphosphorylation, aggregation, and promoted aggregate dissociation, and attenuated pro-inflammatory cytokine. However, HLT showed cytotoxicity at higher concentrations and no neuroprotection against monomeric tau, and the study lacks in vivo validation. The CFPS system provides an excellent environment for drug testing. Wang et al. developed a one-step gold nanoparticle immunochromatographic strip for detecting acetophenetidin in herbal tea, which uses a rationally designed hapten-generated antibody as the recognition element and achieves a visual cut-off value of 160 ng/mL and a quantitative LOD of 1.63 ng/mL within approximately 8 min through competitive lateral flow immunoassay, though the method relies on antibody production and may require further validation for other matrix effects [70].

In summary, these examples illustrate how synthetic biology provides a modular, high-throughput platform for target discovery, drug resistance analysis, and toxicity assessment. Although many of these platforms are not clinical diagnostics in a narrow sense, they represent an important part of the broader diagnostic ecosystem that connects drug discovery, safety assessment, and eventually the introduction of accompanying diagnostics. Meanwhile, Table 2 summarizes the technologies mentioned above for a more intuitive comparison.

## 7. Challenges and Development Bottlenecks

The IVD platform, empowered by synthetic biology, still faces numerous obstacles in transitioning from laboratory prototypes to clinical applications and industrial production. Here, we focus on five major challenges: sample type and preparation system, complexity and stability, obstacles to commercialization and mass production, legal and ethical issues, and challenges in integrated diagnostic workflows.

### 7.1. Sample Type and Preparation

Although synthetic biology-based diagnostic systems have emerged as powerful tools transforming clinical diagnosis, their diagnostic performance is closely tied to clinical sample types and preanalytical preparation protocols, which directly affect the accuracy and reproducibility of detection results and critically impact the reliability of clinical validation. Clinical samples used in such diagnostics exhibit high heterogeneity, including common types such as whole blood, saliva, feces, and tissue biopsies. Their unique matrix components and interfering substances pose significant challenges to the specific recognition of synthetic biological components [71,72].

Specifically, blood-derived components (e.g., hemoglobin, nucleases) can bind to synthetic recognition elements or degrade target biomolecules, thereby increasing the risk of false-negative diagnoses [73]. For fecal samples widely used in the diagnosis of enteric pathogens, a complex matrix requires specialized preprocessing steps to ensure effective recovery of target analytes [74,75]. Notably, synthetic control (SC) samples can effectively standardize the clinical validation process by simulating the characteristics of natural clinical specimens. In contrast, traditional sample extraction methods are usually time-consuming and labor-intensive and prone to introducing variability into clinical validation results [76].

Emerging integrated sample preparation technologies (e.g., paper-based microfluidic devices, ionic liquid-based extraction protocols) can significantly simplify operational procedures and improve the consistency of clinical validation results [77,78]. Ionic liquid-based extraction technology enables rapid, efficient nucleic acid purification within 30 min at room temperature, making it suitable for high-throughput detection and point-of-care (POC) applications. Paper-based microfluidic devices are particularly applicable to resource-constrained clinical settings due to their simple operation and low cost. Conversely, inappropriate sample preparation strategies (e.g., incomplete lysis, insufficient removal of interfering substances) will directly impair the credibility of clinical validation.

The impact of sample-related factors is further amplified during clinical validation, whose core purpose is to evaluate the system’s diagnostic performance in real-world clinical scenarios. A typical case is a synthetic biology-based norovirus biosensor, which exhibited high sensitivity (0.5 fM) in preclinical trials and a strong correlation with RT-qPCR detection results (r = 0.94), but failed to maintain stable performance when non-standardized fecal samples were used for clinical validation [79]. For synthetic biology-based metatranscriptomics diagnostics, synthetic positive controls can effectively improve reproducibility in clinical detection, with a coefficient of variation as low as 0.3%.

In summary, clinical sample types and preanalytical preparation protocols are key factors determining the performance of synthetic biology-based diagnostic systems. Establishing standardized sample-handling guidelines, developing integrated preparation-detection platforms, and applying synthetic control samples are core measures to ensure the reliability of clinical validation, thereby promoting the clinical translation of such diagnostic technologies and ultimately improving the quality of patient diagnosis and treatment.

### 7.2. System Complexity and Stability

Multi-module synthetic diagnostic circuits are often sensitive to environmental fluctuations and prone to functional drift. Several issues are critical.

(i) Temperature and matrix sensitivity. Toehold switches can exhibit spring-loaded behavior, spontaneously opening at higher temperatures and leading to leaky expression and false positives [45]. CFPS reactions lack the homeostatic buffering of living cells; protein synthesis rates depend strongly on substrate concentrations, viscosity, and pH, and are also affected by components present in clinical matrices such as serum and saliva.

(ii) Circuit interference and context dependence. Combining multiple modules, for example, toehold switches, aptamers, and CRISPR effectors, can lead to unanticipated cross-interactions, resource competition, and signal crosstalk, which undermine the predictability of circuit behavior.

Mitigation strategies are beginning to appear. Gupta and Krieg designed a Y-type nucleic acid switch with a “spring-loaded” structure, which not only enhanced the robustness of the nucleic acid signal amplification process but also provided a structural design principle for reducing signal leakage [45]. Banks and Chen conducted a systematic analysis of the dynamic characteristics of CFPS and confirmed that the system’s robustness can be greatly improved by regulating key reaction components, viscosity, and other physical parameters [38,39]. Stone et al.’s study showed that introducing additional inhibitory regulatory mechanisms can stabilize circuit behavior under the influence of growth feedback in living cell systems, and this principle is also applicable to CFPS [80]. Hsu et al. highlighted the importance of cooperative stability in protein complexes, emphasizing its key role in improving system robustness and controllability [81]. Warfel et al. developed a heat-stable CFPS platform, which can also perform its function under field conditions. This study also highlighted the importance of formula optimization for improving system stability [32].

These research results indicate that, to enhance the robustness of the integrated diagnosis system, integrating control theory and materials science is necessary. It includes not only the design of the feedback regulation mechanism, redundant structure, and circuit synergy, but also the refined optimization of the reaction environment and storage form.

### 7.3. Commercialization and Mass Production Barriers

Even if the technical performance is sufficient, the synthetic diagnosis platform will encounter some obstacles in commercialization.

(i) Lack of standardization. Synthetic biological components, such as toehold switches, CRISPR guidelines, and CFPS formulas, are usually tailored to specific designs and are not standardized across laboratories [56]. This heterogeneity makes quality control complicated and limits interoperability. The development of standardized component libraries and interface specifications, similar to electronic component catalogs, will be a necessary condition for scalable manufacturing [82].

(ii) Fragile supply chains. Many platforms rely on custom reagents, including freeze-dried CFPS extracts, specialized oligonucleotides, and custom CRISPR enzymes, which require strict storage conditions and are available from a limited number of suppliers. At the same time, differences in shelf life and batches remain issues that need urgent attention. Shivakumar et al. employed systematic additive screening to enhance the room-temperature stability and productivity of paper- and extracellular-based CFPS, thereby demonstrating the potential of a stronger supply chain [40].

(iii) Limited compatibility with automation. Many prototypes are manually assembled in academic laboratories using small batch freeze-drying and PDMS microprocessing. Industrial-scale manufacturing needs to be transformed into automated-friendly processes, such as injection molding, roll-to-roll printing, and high-throughput reagent dispensing. As envisaged in the biocasting plan, abstract hierarchy and standard workflows can help combine research-level design with automated production pipelines [82].

(iv) Ambiguous regulatory pathways. Hybrid products that combine biological reagents, microfluidic devices, and software, including AI algorithms, do not fit neatly into traditional regulatory categories for IVDs, medical devices, or biologics [57]. This ambiguity can lengthen approval processes and create uncertainty for investors and developers.

To move beyond generic calls for standardization, at least four elements require explicit harmonization. First, the field needs common reference materials, including matrix-matched positive controls and dilution panels, to support cross-platform comparison. Second, biological part activity should be reported using shared performance descriptors, such as leakage, dynamic range, response time, threshold concentration, and lot-to-lot variability, rather than narrative descriptions alone. Third, benchmark assay panels should be established to compare CFPS, toehold, CRISPR, and hybrid workflows under identical sample and readout conditions. Fourth, validated control architectures should be routinely incorporated, including internal amplification controls, negative matrix controls, and orthogonal confirmation modules. At the platform level, a standardized data representation format for circuit behavior and a “diagnostic BioBrick” concept for interoperable sensing, processing, and output modules would substantially improve reproducibility, comparability, and manufacturability.

### 7.4. Legal and Ethical Issues

Synthetic biology-based diagnostics raise legal and ethical questions that extend beyond those encountered with conventional assays.

(i) Regulatory classification and oversight. Existing IVD regulations are usually organized around composition and intended use, making it difficult to classify cellless synthetic circuits, engineering biological diagnostics, and integrated biological digital platforms. For example, in Europe, the regulatory systems for genetically modified organisms and traditional IVD products cannot be directly applied to synthetic cell diagnostic technology, creating a regulatory gap and potentially delaying the clinical transformation of related technologies [57].

(ii) Dual-use and biosecurity risks. While the programmability of diagnostic technology brings beneficial value, it also has the risk of abuse. Such technologies may be used to detect or mask specific biological agents, thereby evading regulation, and even facilitating the research and development of pest structures [83,84]. In view of these potential risks, we urgently need to establish a more comprehensive biosafety framework, implement a transparent risk assessment mechanism, and strengthen the ethical self-discipline of the industry throughout the entire process of technological research and commercialization.

(iii) Data privacy and ownership. With the gradual integration of diagnostic technology into the Internet of Things (IoT) medical architecture, coupled with the application of cloud analysis technology and long-term patient tracking, data privacy protection, data ownership definition, and data secondary use specification have become core issues to be addressed. Nišević et al. have discussed the legal and ethical issues of medical synthetic data, which are closely related to cloud-connected synthetic diagnostic platforms [85]. Ensuring informed consent, secure data processing, and transparent governance are crucial to maintaining patient trust.

An additional regulatory challenge emerges when the diagnostic assay becomes inseparable from the software layer that interprets, updates, or clinically contextualizes its signal. In such cases, the regulatory object is no longer only the wet-lab assay, chip, or cartridge, but also the algorithmic component that may function as software as a medical device or as software embedded within a medical device. For next-generation synthetic biology-enabled IVDs, this means that analytical validation, clinical validation, cybersecurity, transparency, post-market monitoring, and change-management strategy may need to be assessed together rather than in isolation. In particular, adaptive or continuously updated AI models raise regulatory questions distinct from those of static diagnostic assays, because model modifications can change performance after deployment. Future translation will therefore require early co-design of assay chemistry and software governance, so that biological innovation is aligned with emerging frameworks for AI-enabled medical software rather than delayed by them [86].

### 7.5. Challenges in Integrated Diagnostic Workflows

Despite the remarkable advantages of synthetic biology in core aspects such as molecular recognition, signal transduction, and the construction of detection devices, numerous key bottlenecks in the integrated diagnostic workflow remain to be systematically addressed. Components in clinical samples, including nucleases, proteases, mucus, and inhibitors from biological matrices, easily interfere with CFPS, conformational changes in nucleic acid switches, and CRISPR reaction efficiency. Mismatches in temperature, buffer systems, reaction kinetics, and energy substrates among cascaded modules reduced signal amplification efficiency. Incompatible interfaces between biological components and carrier materials (e.g., paper-based substrates, microfluidics), non-specific adsorption, and cross-contamination further impair system stability and reproducibility. Meanwhile, insufficient automation throughout the workflow, aggravated signal crosstalk and background noise, lack of unified quality control standards, and unclear regulatory pathways collectively restrict the clinical translation of laboratory prototypes into POCT applications.

To overcome these obstacles, potential strategies include engineering synthetic biology chassis with high matrix tolerance, developing one-step integrated systems compatible with isothermal and universal buffer conditions, optimizing material-bio interfaces to minimize non-specific adsorption, designing built-in logic gating and internal reference calibration systems to improve signal-to-noise ratio, promoting fully automated integration via microfluidics and lyophilized reagents, and establishing standardized quality control and validation frameworks spanning from compositional elements to integrated systems. These approaches will systematically enhance the robustness, reliability, and practicality of integrated diagnostic workflows, thereby providing critical support for the clinical translation and large-scale application of synthetic biology-driven in vitro diagnostic technologies. Ma et al. [87] proposed a strategy employing multi-armed RNA linkers in combination with RNA activation elements with relative sequence independence, enabling the implementation of AND/OR logic gates in a paper-based cell-free system. They further achieved target recognition in saliva samples and amplified RNA templates, demonstrating a route to improving anti-background interference and readout reliability via logic gating. AbdElFatah et al. [88] integrated nanoplasmonic surfaces into automated microfluidic cartridges to achieve equivalent acceleration via hot-electron injection, which markedly reduced the time required for amplification and colorimetric quantification. Combined with machine learning and device-integrated readout, this work highlights the paradigm of material functionalization, microfluidic automation, and rapid readout.

## 8. Future Development Directions

The frontier of synthetic biology-enabled IVD is shifting from isolated proof-of-concept demonstrations toward integrated systems intended for use in clinics, communities, and homes. We highlight three interrelated directions: technological integration, platform standardization, diagnostics-as-a-service, and clinical adaptation coupled with real-world validation.

### 8.1. Technological Integration: AI, Multimodal Readouts, and Wearable/Implanted Systems

AI should not be treated as a single enabling label, but as a set of distinct functions across the synthetic biology-enabled IVD workflow. First, AI can support in silico design of biological parts by predicting toehold switch folding behavior, guide-RNA efficiency, off-target risk, or circuit-level dose–response properties, thereby reducing empirical screening burden. Second, AI can enhance signal processing by integrating fluorescence, colorimetric, electrochemical, or imaging outputs from complex chips and compensating for noise, drift, and matrix-dependent variation. Metsky et al. [46] confirmed that large-scale targeted optimization datasets significantly improve CRISPR-based mutation detection performance. Combining fluorescence, colorimetric, and electrochemical multimodal readout strategies further enhances detection robustness and reliability. Third, AI can contribute to clinical decision support by contextualizing diagnostic readouts with patient metadata, disease prevalence, or longitudinal trends. These three roles differ substantially in their data requirements and validation logic: design models require large, well-annotated sequence–function datasets; signal-processing models require device-specific calibration and robustness to domain shift; and decision-support models require clinically curated cohorts and careful evaluation of interpretability, bias, and generalizability. A mature future framework should therefore discuss AI in these separate layers rather than as a generic route to “higher sensitivity and specificity” [89].

Latest synthetic biology tools optimize sensor performance for wearable/implantable applications. For example, Chen et al.’s split intron-mediated trans-splicing denoiser reduces leakage expression by >21-fold and boosts dynamic range by 9-fold (without sacrificing sensitivity), improving signal-to-noise ratios for long-term stable sensors [90]; AI integration enables adaptive biomarker detection by optimizing response thresholds based on real-time data. Wan et al. [91] reported cascaded amplifying circuits (5000-fold detection limit improvement, 750-fold output increase for toxic metal sensors) that enhance low-abundance biomarker detection in wearables. The AGATHA (Atypical gRNA-activated Transcription Halting Alarm) biosensor, based on reprogrammed tracrRNAs, enables signal-amplified specific RNA detection for wearable pathogenic RNA monitoring [92].

### 8.2. Platform Standardization and Diagnostics-as-a-Service

Platform standardization should proceed at three interconnected levels: parts, performance reporting, and system interfaces. At the parts level, curated libraries of validated sensors, reporters, CRISPR components, and CFPS formulations are needed, together with metadata on target class, matrix compatibility, storage stability, and failure modes. At the performance-reporting level, the field would benefit from a common benchmarking framework that records not only the limit of detection and assay time, but also leakage, dynamic range, reporter visibility, batch robustness, and behavior in representative clinical matrices. For example, Lopreside et al. [93] systematically compared the sensing performance of eight mainstream genetic reporter genes in both whole-cell and cell-free systems, and identified NanoLuc as the optimal high-sensitivity and fast-response reporter element, providing a better unified benchmark for the rational selection of reporter genes in synthetic biosensors. At the system level, interoperable interfaces between recognition, amplification, output, and microfluidic modules would enable a true plug-and-play “diagnostic BioBrick” architecture. Such an approach would transform synthetic biology-enabled IVD from a collection of bespoke prototypes into a transferable engineering platform that supports automated design–build–test–learn cycles, distributed manufacturing, and more efficient regulatory evaluation [82].

Advanced synthetic biology tools enrich standardized part libraries: Shen et al.’s directed evolution-optimized PbrR-based whole-cell biosensor (LOD = 0.045 μg/L, stable in real water/tea samples) serves as a standard lead detection BioBrick [94]; Lee et al.’s CFPS system (heavy metal detection via personal glucose meter) provides standardized plug-and-play components (e.g., metal-responsive transcription factors, invertase reporters) [95]; Gao et al.’s multi-level regulatory circuits (DNA/RNA/protein-level elements) offer a modular framework for customizable sensor design, enhancing platform flexibility [96].

This infrastructure supports a diagnostics-as-a-service model, delivering customizable solutions to clinics, public health agencies, and individuals. Kim et al. developed an IoT-integrated automatic POC molecular diagnostic device, demonstrating the potential for remote/home-based synthetic diagnosis [97]. Banerjee et al. reported PINAT (single-chamber, minimal-user-intervention isothermal nucleic acid testing) for SARS-CoV-2/influenza sample-to-result detection [98]. Integrating synthetic modules CFPS for expanded small-molecule detection, Liu et al.’s AGATHA for specific nucleic acid detection) into these standardized platforms is critical for widespread adoption, especially in resource-limited settings [92].

### 8.3. Clinical Adaptation and Real-World Validation

The ultimate value of synthetic biology-driven IVD lies in its impact on patient prognosis, medical workflows, and public health responses. Realizing this value requires three key efforts: (1) establishing tailored regulatory pathways with exclusive classification guidelines for cell-free and CRISPR-based diagnostics; (2) integrating diagnostic technologies into real-world data systems (e.g., disease registries) to accumulate routine clinical effectiveness data; (3) prospectively comparing synthetic biology platforms with existing clinical standards across diverse patient groups and scenarios.

Advanced synthetic biology tools address sensitivity/specificity/stability challenges for clinical translation: Shen et al.’s PbrR-E3 biosensor shows graphite furnace atomic absorption spectrometer (GFAAS)-consistent results in tea samples (food safety application) [94]; Chen et al.’s uric acid/lactate biosensors accurately quantify physiological biomarkers (metabolic disease diagnosis) [90].

Clinical adaptation will also require co-validation of biological and digital components in real-world settings. For platforms that incorporate algorithm-assisted interpretation, prospective multicenter studies should evaluate not only analytical sensitivity and specificity, but also calibration drift, inter-operator reproducibility, failure modes in heterogeneous samples, and performance stability after software updates. Real-world evidence frameworks, interoperability with hospital information systems, and predefined post-market monitoring plans will be especially important for assays intended for decentralized or home use. In this sense, the path to translation is not merely a matter of improving assay chemistry, but of establishing an evidence ecosystem in which the sensing module, device hardware, software layer, and clinical workflow are validated as an integrated diagnostic system.

## 9. Conclusions

Synthetic biology is enabling a new generation of programmable biosensing platforms for rapid and decentralized IVD. By providing standardized, programmable modules for molecular identification, signal processing, and system integration, synthetic biology has enabled a transformation from static, single-analyte analysis to a flexible, reconfigurable diagnostic platform.

In this review, we outline the conceptual basis and historical background of synthetic biology and IVD, summarize the key core modules, including the CFPS system, toehold switches, aptamer or ribose switch sensor, CRISPR-Cas detection platform, microfluidic control integration, and focus on the representative applications in rapid infectious disease detection, cancer biomarker detection, drug metabolism or toxicity screening, etc. We also studied major challenges related to system stability, standardization, supply chain, automation, supervision, biological security, and data privacy. And put forward the future development direction centered on technology integration, platform standardization, and clinical adaptation.

Looking ahead, three tasks appear particularly important. First, synthetic diagnostic circuits must be engineered to remain robust in the face of real-world variability. Second, it is necessary to establish a platform compatible with standardization and automation to reduce obstacles to manufacturing and deployment. Third, high-quality clinical and real-world evidence must be produced to support the approval and reimbursement by regulatory authorities. If these challenges can be solved, the IVD system based on synthetic biology can be well developed into an intelligent, embedded diagnostic ecosystem, thereby supporting personalized medical care, rapid response to epidemics, and enhancing biosafety in an increasingly interconnected world.

## Figures and Tables

**Table 1:** Key performance indicators of core technological modules.

Detection Technology	Limit of Detection (Typical Range)	Detection Time (Typical Range)	Core Advantages	Main Limitations	Ref.
CFPS	1 pM −100 fM	30 min −2 h	1. Rapid preparation of diagnostic reagents such as antigens and enzymes without cell culture; 2. Tunable reaction system, compatible with various reporter molecules; 3. Lyophilizable and fixable, suitable for paper-based/microfluidic platforms and on-site detection; 4. Compatible with a cell-free environment and low biosafety risk.	1. Reaction is sensitive to temperature and ionic strength, easily interfered with by components in clinical matrices; 2. Higher reagent cost per reaction compared with traditional enzymatic detection; 3. Rehydration efficiency after lyophilization is prone to batch variation, and reproducibility needs improvement.	[30,31,33]
Toehold switch	1 fM −1 pM (down to aM level when combined with amplification technology)	20 min −1.5 h	1. High programmability, rapid adaptation to different target RNAs/miRNAs; 2. No need for expensive instruments, enabling visual detection; 3. Good compatibility with CFPS, capable of constructing integrated detection circuits; 4. High specificity, distinguishable for single-nucleotide variations.	1. Prone to spontaneous unwinding at high temperatures, leading to false positives; 2. Sensitive to the secondary structure of target RNA, high design difficulty; 3. Poor LOD (limit of detection) without amplification, relying on auxiliary amplification technology.	[42,43,44]
Aptamer /Riboswitch	1.63 ng/mL −166 nM (varies by target type)	30 min −2 h	1. Targetable to non-nucleic acid targets such as proteins and small molecules, complementing the gap of nucleic acid detection; 2. High affinity and specificity, customizable screening; 3. Easy integration with lateral flow strips and electrochemical sensors, simple operation.	1. Complex aptamer screening process, time-consuming; 2. Greatly affected by matrix composition and temperature, insufficient structural stability; 3. Off-target effects easily lead to false positives, requiring strict optimization.	[47,49,50]
CRISPR-Cas (Cas12/13/14)	1 copy/μL −1 aM (varies by whether combined with amplification)	15 min −1 h	1. Extremely high sensitivity, enabling single-copy detection when combined with RPA/LAMP amplification; 2. High specificity, distinguishable for single nucleotide differences in homologous sequences; 3. Simple operation, no need for precision instruments, suitable for on-site POCT; 4. Capable of multi-target parallel detection.	1. Amplification-dependent systems are prone to contamination, leading to false positives; 2. Higher LOD without amplification, difficult to detect low-abundance targets; 3. Large batch variation in enzyme preparations, high quality control difficulty; 4. Mostly qualitative/semi-quantitative detection, with weak quantitative capability.	[52,53]
Microfluidic integrated	1 aM −100 fM (varies by integrated modules)	20 min −1.5 h	1. Integrating the entire process of sample processing, amplification, and detection, with a high degree of automation; 2. Low reagent consumption, reduced cost, and low contamination risk; 3. Capable of multi-module synergy, improving detection efficiency; 4. Miniaturized design, suitable for point-of-care and primary on-site detection.	1. Complex chip preparation process, high difficulty in large-scale production; 2. Easy leakage and cross-contamination of fluids in multi-compartment; 3. Easy adsorption and inactivation of biological components on chip materials, insufficient stability; 4. High equipment R&D cost, difficult to popularize.	[58,59]

**Table 2:** Comparison of representative application technologies.

Technology Name	LOD	Detection Time	Detection Technology Type	Current Development/Clinical Stage	POC IVD?	Ref.
Cell-free toehold switch sensor for hepatitis A virus type I (HAV I)	5 nM (without amplification), 1 pM (with NASBA amplification)	~2 h (cell-free reaction); ~4 h with NASBA (2 h amplification + 2 h detection)	CFPS + toehold switch RNA sensor	Preclinical laboratory validation	Yes	[60]
C-TEC-CRISPR/Cas14a	50 fM (Naked eye); 20.71 fM (UV-vis); 257 fg/μL (Genomic DNA)	90 min	TWJ + EXPAR + CRISPR/Cas14a	Preclinical	Yes	[61]
CRISPR-Cas13a coupled with RPA for rapid AMR detection	1 aM (two-step format), 10 aM (one-tube format), 10 fM (lateral flow dipstick)	5 min (one-tube), 40 min (two-step)	Nucleic acid amplification + CRISPR	Preclinical laboratory validation	Yes	[62]
Cas12a-UPTLFA portable nucleic acid detection platform	3 aM (purified DNA), 10^2^ CFU/100 μL (spiked blood)	~1.3 h total (18 min RPA, 45 min Cas12a reaction, 15 min LFA readout)	CRISPR-Cas + lateral flow immunoassay	Preclinical laboratory validation	Yes	[63]
Synthetic protein protease sensor platform	α-HIS: 20 ng; α-GFP: 8 ng	1.5 h	Synthetic protein sensor + ELISA/fluorescence	Preclinical	Yes	[64]
Transmembrane DNA logic nanodevices (TDLNs) based on a DNA tetrahedron and localized CHA	201 pM (MUC1), 28.3 pM (miR-21)	~1 h (in vitro), 2 h (cell incubation and imaging)	Fluorescence imaging (FRET) + localized catalytic hairpin assembly (CHA) + DNA logic gate (AND)	Preclinical	No	[65]
Split-Cas9-based synthetic logic circuits	Not reported (qualitative logic sensing)	48–72 h	Split-Cas9 + AND logic gate	Preclinical	No	[66]
Antigen-dependent inducible T cell reporter system (SNIPR-PET)	Not specified (qualitative/semi-quantitative imaging)	In vitro: 48–72 h; In vivo: 3–10 d (peak at day 8)	CRISPR-Cas9 + fluorescence imaging	Preclinical	No	[67]
Yeast two-hybrid assay coupled with biochemical validation for screening inhibitors targeting *M. tuberculosis* FtsZ–SepF interaction	IC_50_ = 20.42 μmol/L (T0349); MIC against Mtb H37Rv = 2 μg/mL	~48–72 h (yeast screening); ~24 h (MIC determination)	SPR + CRISPRi	Preclinical	No	[68]
Integrated cell-free and cell-based screening platform targeting tau pathology	Not reported (qualitative/relative efficacy evaluation)	4–24 h	ThT + ICC	Preclinical	No	[69]
One-step ultra-sensitive immunochromatographic strip for acetophenetidin detection	1.63 ng/mL (quantitative), 160 ng/mL (visual cut-off)	8 min (3 min incubation + 5 min chromatography)	Lateral flow immunochromatographic + gold nanoparticle labeling	Preclinical/laboratory validation	Yes	[70]

## Data Availability

No new data were created or analyzed in this study.
